# The Novel Type 1 Fimbriae FimH Receptor Calreticulin Plays a Role in *Salmonella* Host Specificity

**DOI:** 10.3389/fcimb.2017.00326

**Published:** 2017-07-19

**Authors:** Krzysztof Grzymajlo, Maciej Ugorski, Jaroslaw Suchanski, Anna E. Kedzierska, Rafal Kolenda, Anna Jarzab, Agnieszka Biernatowska, Peter Schierack

**Affiliations:** ^1^Department of Biochemistry and Molecular Biology, Faculty of Veterinary Sciences, Wrocław University of Environmental and Life Sciences Wrocław, Poland; ^2^Ludwik Hirszfeld Institute of Immunology and Experimental Therapy, Polish Academy of Sciences Wrocław, Poland; ^3^Faculty of Environment and Natural Sciences, Institute of Biotechnology, Brandenburg University of Technology Cottbus-Senftenberg Senftenberg, Germany; ^4^Laboratory of Cytobiochemistry, Faculty of Biotechnology, University of Wrocław Wrocław, Poland

**Keywords:** *Salmonella*, host specificity, type 1 fimbriae, FimH, adhesion, calreticulin, receptor

## Abstract

It was suggested that minor differences in the structure of FimH are most likely associated with differences in its adhesion specificities and may determine the tropism of various *Salmonella* serovars to different species and tissues. We have recently shown that FimH adhesins from host-adapted serovars, e.g., *Salmonella* Choleraesuis (*S*Ch), bind to other glycoprotein receptors compared to FimH from host-unrestricted *Salmonella* Enteritidis (*S*E). Here we identify porcine calreticulin expressed by swine intestinal cells as a host-specific receptor for *S*Ch FimH adhesin, suggesting that such an interaction may contribute to *S*Ch host specificity. Calreticulin was identified by 2D electrophoresis and mass spectrometry as a glycoprotein that was bound specifically by recombinant *S*Ch FimH protein, but not by FimH from *S*E. The functionality of calreticulin as a specific receptor of *S*Ch FimH adhesin was further confirmed by adhesion and invasion of mutated strains of *S*Ch carrying different variants of FimH proteins to IPEC-J2 cells with overexpression and silenced expression of calreticulin. It was found that *S*Ch carrying the active variant of FimH adhered and invaded IPEC-J2 cells with calreticulin overexpression at significantly higher numbers than those of SCh expressing the non-active variant or *S*E variant of FimH. Moreover, binding of *S*Ch carrying the active variant of FimH to IPEC-J2 with silenced calreticulin expression was significantly weaker. Furthermore, we observed that *S*Ch infection induces translocation of calreticulin to cell membrane. All of the aforementioned results lead to the general conclusion that *Salmonella* host specificity requires not only special mechanisms and proteins expressed by the pathogen but also specifically recognized receptors expressed by a specific host.

## Introduction

Adhesion to host tissues is a crucial step during bacterial pathogenesis. Initial bacteria attachment often involves a specific interaction between specialized protein structures presented at the surface of bacteria cells and receptors presented on the host cell surface. *Salmonella* establish various strategies to adhere to host tissues by expressing an enormous number of both fimbrial and non-fimbrial adhesins, which are sometimes directly linked with the outcome of bacterial infection (Wagner and Hensel, 2011). One of the broadly expressed and well-characterized fimbrial structures are type 1 fimbriae, encoded by the *fim* operon. These filamentous organelles present on the bacteria surface, are composed primarily of structural protein FimA, however, lectin-like protein, named FimH, is directly involved in binding to high-mannose oligosaccharides carried by surface glycoproteins of eukaryotic cells (Krogfelt et al., 1990; Jones et al., 1995). Type 1 fimbriae play an important role in these initial stages of infection (Ewen et al., 1997; Dibb-Fuller et al., 1999; Dibb-Fuller and Woodward, 2000; Naughton et al., 2001) and can contribute to the host tissue tropism of *Salmonella* serovars (Baumler et al., 1997; Humphries et al., 2001; Edwards et al., 2002). There is a growing body of literature that recognizes that minor differences in the structure of FimH are most likely associated with differences in adhesion specificities and may determine the tropism of various *Salmonella* serovars to different species and tissues (Boddicker et al., 2002; Guo et al., 2009; Kisiela et al., 2012; Kuzminska-Bajor et al., 2012). Our previous study showed that FimH adhesins from host-adapted serovars - *Salmonella* Choleraesuis, *Salmonella* Abortusovis and *Salmonella* Dublin - bind to membrane proteins of approximately 55 kDa expressed by pig, sheep, and cattle enterocytes, respectively. In contrast, FimH protein from host-unrestricted *Salmonella* Enteritidis binds to glycoproteins of approximately 130 kDa present on the surface of these cells (Grzymajlo et al., 2013). Therefore, our data suggest the existence of specific receptors expressed by host cells, which are selectively recognized by allelic variants of FimH adhesins expressed by *Salmonella* serovars with different host specificities. It was shown before, using human, bovine and porcine intestinal epithelial cells, that FimH protein variant from *S*. Choleraesuis binds significantly better to porcine enterocytes when compared to other allelic variant, and mediated poor binding to human intestinal epithelial cells (Yue et al., 2015). Based on those results, as well as on our findings, we decided to further explore this model.

Despite the different types of *Salmonella* adhesins described to date (Wagner and Hensel, 2011), there is only limited knowledge regarding host receptors involved in *Salmonella* infections. As far as type 1 fimbriae and FimH adhesin are concerned, there were only a few examples of putative receptors, such as carcinoembryonic antigens (Leusch et al., 1991), a 60 kDa glycoprotein from the rat brush border membrane (Ghosh et al., 1996), plasminogen (Kukkonen et al., 1998) or cystic fibrosis transmembrane conductance regulator, a serovar specific receptor for *S*. Typhi (Pier et al., 1998; Lyczak and Pier, 2002). Moreover, there is only one well-characterized receptor for FimH adhesin to date: glycoprotein 2 expressed on M cells (Hase et al., 2009).

Enterocytes represent up to 95% of total intestinal cells, and its apical surface bears a wide range of glycoconjugates that may act as potential receptors for bacterial adhesins. Established enterocyte cell lines express intestinal membrane components and, therefore, can act as a suitable model for host-pathogen interactions in the initial phases of bacterial pathogenesis (Brosnahan and Brown, 2012). In our previous study (Grzymajlo et al., 2013), we found that the IPEC-J2 cell line (Schierack et al., 2006), a widely described porcine intestinal cell model, expresses a protein specifically recognized by *S*. Choleraesuis FimH. Here, we identify this 55 kDa FimH receptor as calreticulin (CRT) and define it as a major interaction partner for FimH expressed by host-adapted *S*. Choleraesuis. Furthermore, we analyzed the role of the novel discovered receptor in FimH-mediated adhesion and invasion of *S*. Choleraesuis to swine enterocytes in a host-specific manner as well as the impact of *Salmonella* infection on the expression and localization of the receptor. This study provides new insights into host specificity of *Salmonella*, indicating for the first time host and pathogen protein interactions directly involved in that phenomena.

## Materials and methods

### Bacterial strains and plasmids

All *Salmonella* mutants were derived from *S*. Choleraesuis strain 6150 isolated from porcine feces (Isolated in Schierack Lab, Institute of Biotechnology, Faculty of Environment and Natural Sciences, Brandenburg University of Technology Cottbus-Senftenberg, Großenhainer Str. 57, 01968, Senftenberg, Germany). The bacterial strains and plasmids used in this study are listed in Table 1.

Table 1Bacterial plasmids and strains used in this study.

**Table:** 

**Plasmid**	**Characteristic**	**References**
pKD46	Λ Red recombinase expression plasmid	The Coli Genetic Stock Center, Yale University, USA
pCP20	FLP recombinase plasmid	The Coli Genetic Stock Center, Yale University, USA
pACYC177	Cloning vector	ATCC® 37031™
pACYC177/C63	FimH from *S*. Choleraesuis inactive variant (63G) cloned into pACYC177	This study
pACYC177C	FimH from *S*. Choleraesuis active variant (63V) cloned into pACYC177	This study
pACYC177/E	FimH from *S*. Enteritidis active variant cloned into pACYC177	This study
pET22b/CextfimH	Expression vector encoding FimH active variant from *S*. Choleraesuis	Grzymajlo et al., 2013
pET22b/C63extfimH	Expression vector encoding FimH inactive variant (V63G) from *S*. Choleraesuis	This study
pET22b/EextfimH	Expression vector encoding FimH active variant from *S*. Choleraesuis	Grzymajlo et al., 2013

**Table:** 

**Strains**	**Strain tag**	**Characteristic**	**Reference**
*S*. Choleraesuis Δ*fimH*	SCΔ*fimH*	*S*. Choleraesuis 6150 with *fimH* knockout	This study
*S*. Choleraesuis Δ*fimH*	SCΔ*fimH/*Empty	SCΔ*fimH* carrying pACYC177	This study
*S*. Choleraesuis Δ*fimH*/Ch-non-binding	SCΔ*fimH*/C63	SCΔ*fimH* carrying pACYC177/C63	This study
*S*. Choleraesuis Δ*fimH*/Ch-high	SCΔ*fimH*/C	SCΔ*fimH* carrying pACYC177/C	This study
*S*. Choleraesuis Δ*fimH*/En	SCΔ*fimH*/E	SCΔ*fimH* carrying pACYC177/E	This study

### Generation of *fimH* gene deletion mutant

The deletion mutant was generated according to the Datsenko-Wanner method with minor modifications (Datsenko and Wanner, 2000). Briefly, electro-competent bacteria were transformed with pKD46 plasmid, grown at 30°C for 2 h with shaking and plated on agar with ampicillin (100 μg/ml) for 24 h at 30°C. Next, bacteria bearing pKD46 plasmid were used for preparation of electro-competent cells according to protocol described in Khetrapal et al. (2015) and transformed with PCR products containing chloramphenicol resistance cassette (Cam) and *fimH* gene homologous extensions. Bacteria, in which Cam resistance cassette was successfully introduced into *fimH* gene locus were selected on LB agar plates with Cam (final concentration of Cam: 10 μg/ml) and later successful introduction of resistance cassete into *fimH* gene locus was confirmed by colony PCR using following primers: fimH_Sal_For (ATCCAGTGGGGAGAGGG), fimH_Sal_Rev (GAGTTGGCCTGACTCAGC),O1535 fimH_Sal_qPCR_rev (CTTTGCCGCTGTAGCTAATGG) and C1 (Datsenko and Wanner, 2000). In the next step, Cam resistance cassette was removed using pCP20 plasmid and successful removal of Cam resistance cassete from *fimH* gene locus was confirmed by colony PCR and Sanger sequencing.

### Cloning of *fimH* gene alleles into the pACYC177 plasmid

*FimH* gene sequences were amplified with PCR using the following primers: FimHpACYCfor- ACATGGATCCTTGACAATTAATCATCGGCTCGTATAATGTGTGGAG GAGGACAGCTATGAAAATATACTCAGCGCTATTG and FimHpACYCrev- ACATGGATCCTTAATCATAATCGACTCGTAGATAG. The resulting DNA fragments were amplified with *Phusion* High-Fidelity DNA Polymerase (Thermo- Scientific) according to the manufacturer's protocol. Briefly, 50 μl of the reaction mix contained: genomic DNA isolated from *S*. Choleraesuis isolate no. 1656/04 (endogenous CFimH) or *S*. Choleraesuis 1204 (endogenous C63FimH) or *S*. Enteritidis isolate no. 327 (endogenous EFimH) (1 μl), *Phusion* HF buffer (Thermo Scientific), MgCl_2_ (at final concentration of 1.5 mM), 1 U of *Phusion* High-Fidelity DNA Polymerase (Thermo Scientific), 0.2 μM of each primer and 0.2 mM dNTPs mix. PCR was started with initial denaturation (30 s, 98°C), then 35 cycles of denaturation (5 s, 98°C), annealing (15 s, 58°C), elongation (40 s, 72°C) and final elongation (72°C, 5 min). PCR products were cloned into the pACYC177 plasmid. FimH protein sequence variants used in this study are presented in Table 2.

**Table 2:** FimH protein sequence variants.

**AA Position**	**57**	**63**	**89**	**137**
**Alternative AA position**	35	41	67	115
CFimH (*S*. Choleraesuis)	**L**	**V**	**R**	**K**
C63FimH (*S*. Choleraesuis)	**L**	**G**	**R**	**K**
EFimH (*S*. Enteritidis)	**P**	**V**	**Q**	**M**

### Generation of *Salmonella* isogenic model

*Salmonella* Choleraesuis Δ*fimH* was electroporated with pACYC177/C, pACYC177/C63, or pACYC177/E plus empty pACYC177 plasmid as negative control. The resulting *Salmonella* strains were named SCΔ*fimH*/C, SCΔ*fimH*/C63 (*S*. Choleraesuis FimH mutant V63G), SCΔ*fimH*/E, and SCΔ*fimH/*Empty, respectively.

### *Salmonella* mutant binding activity

To measure the binding abilities of *Salmonella* mutants, we applied 100 μl of bacteria (CFU 5 × 10^7^/ml) to each well of 96-well plates coated with polyclonal rabbit anti-FimH antibody (Kisiela et al., 2005) and glycoprotein standards and then incubated them for 2 h at room temperature followed by three washing steps with PBS. Attached bacteria were fixed with 50 μl of 4% PFA (paraformaldehyde) in PBS for 30 min at 4°C, washed three times with 100 μl of PBS and stained with 50 μl of propidium iodide (10 μg/ml in ddH_2_O) for 15 min at room temperature. Plates were read using the VideoScan module (Rodiger et al., 2013; Schierack et al., 2015). Three independent experiments with three repetitions for each dilution were prepared and measured (Figure S1).

### Cells and cell culture

Intestinal epithelial IPEC-J2 cells from newborn porcine (Schierack et al., 2006) were cultured in DMEM supplemented with 10% fetal calf serum (FCS, Invitrogen, Carlsbad, USA), 2 mM glutamine and antibiotics. The mouse small intestine cell line (ICE-1) was a kind gift from InScreenex (InSCREENeX, Braunschweig, Germany) (Schwerk et al., 2013). Cells were cultured in a defined medium supplied by the manufacturer.

### Construction of expressing vector

For the generation of a vector expressing calreticulin, initially, IRES sequence derived from the pWP1 vector (kindly provided by Dr. D. Trono, École Polytechnique Fédérale de Lausanne, Switzerland) and a DNA cassette containing puromycin N-acetyl-transferase (PAC) cDNA from a pPUR vector (Clontech, Terra Bella Avenue Mountain View, CA, USA) were cloned into the pRRL-cPPT-CMV-X2-PRE-SIN vector. To obtain porcine CRT cDNA sequence, we isolated total RNA from IPEC-J2 cells, and a cDNA library was generated with a transcriptor first strand cDNA synthesis kit using an oligo (dT) primer (Roche). CRT cDNA was amplified by PCR with the primers 5′GAACGCGTATGCTGCTCCCAGTCCC′ and 5′GACTCGAGCTACAGCTCATCCTTGGCC3′ containing restriction sites for MluI and Xho1, respectively, and was cloned into the pRRL-CMV-IRES-PURO vector. This vector was named pRRL-CALR-IRES-PURO.

To produce virus, we used lentiviral vectors derived from the human immunodeficiency virus (HIV-1). *Polyethylenimine*-mediated (Sigma-Aldrich, Buchs, Switzerland) transient co-*transfection was mediated using* packaging LentiX 293T cells (Clontech, Cat. No. 632180) at 50–60% confluence with 20 μg of pRRL-CMV-CALR-IRES-PURO or pRRL-CMV-IRES-PURO, 10 μg of pMDL-g/p-RRE, 5 μg of pRSV-REV, and 5 μg of pMk-VSVG (D. Trono, École Polytechnique Fédérale de Lausanne, Switzerland). Viral supernatant was collected 48 h after transfection and was then clarified through a 0.45 μm pore size filter (Millipore, Billerica, MA, USA). Immediately afterwards, the virus-containing supernatant was concentrated 100X on an Amicon Ultra-15K:100.000 (Millipore, Billerica, MA, USA).

### Construction of silencing vector containing small hairpin RNA (shRNA/CRT)

The pLVTHM/PURO-shCRT silencing vector was obtained essentially as described previously (Solatycka et al., 2012). pLVTHM/GFP lentiviral vector was kindly provided as part of the lentivirus system by Dr D. Trono (Ecole Polytechnique Fédérale de Lausanne, Switzerland). Briefly, shRNA/CRT targeted the GGATTGAATCCAAACACAAAC fragment of the CRT mRNA (NP_001167604). The DNA cassette consisted of the H1 promoter, and the shDNA/CRT sequence was cloned into the pLVTHM/PURO vector. To obtain the pLVTHM/PURO vector, we replaced GFP cDNA by the DNA cassette containing the puromycin N-acetyl-transferase (PAC) cDNA from a pPUR vector (Clontech, Terra Bella Avenue Mountain View, CA, USA). Lentivirus-induced CRT silencing was obtained essentially as described above with the exception of the lentivectors used: 20 μg of pLVTHM/PURO-shCRT or pLVTHM/PURO co-transfected with the packaging plasmids, 15 μg of psPAX2 and 5 μg of pMD2.G (kindly provided by Dr D. Trono, Ecole Polytechnique Federale de Lausanne, Switzerland), into the LentiX 293T cell line.

### Construction of a cell line expressing calreticulin

IPEC-J2 or ICE-1 cells were infected with concentrated virus stock (both silencing and expressing) by centrifugation (2,460 × g) at 23°C for 2.5 h. After overnight incubation, the medium was replaced, and after the next 48 h, cells were selected in 1 μg/ml of puromycin.

Generated cell lines were named IPEC-PURO (control line for overexpression), IPEC-CRT (overexpression), ICE-PURO (control line for ectopic expression), ICE-CRT (ectopic expression), IPEC-shPURO (control line for silencing), and IPEC-shCRT (silencing). Growth rate of generated cell lines was tested by SRB assay.

### Proliferation assay (SRB assay)

The cells (1 × 10^4^) were grown in 96-well plates (Sarstedt, Germany) in complete DMEM. After every 24 h, they were fixed by the addition of 200 μl of 10% trichloroacetic acid for 30 min at 4°C, washed with water, and dried. Fixed cells (after 24, 48, 72, and 96 h) were incubated with 0.4% sulforhodamine B (SRB, Sigma) in 1% acetic acid for 20 min in RT. After washing with 1% acetic acid, the protein-bound dye was extracted with 10 mM Tris for 1 h at 37°C. The absorbance at 562 nm was measured in plate reader “Quanta” (Bio-tek). Data were presented as the means ± SD from three independent assays.

### Quantitative PCR (qPCR)

Total RNA was isolated from 10^6^ cells using RNeasy plus mini Kit (Qiagen) according to the manufacturer's recommendations. First-strand cDNA was synthesized using the iScript (Bio-Rad). The relative amounts of mRNA were quantified by qPCR using the CFX thermocycler (Bio-Rad) and SYBR-green (Life Technologies). *Actb* was used as the reference gene, and all primers used are listed in Table 3. The comparative Cq method was used for relative quantification of gene expression. All experiments were performed at least four times, and triplicate samples were analyzed in each experiment to confirm the accuracy and reproducibility of the qPCR. Fold change was measured over unstimulated cells set at 1.

**Table 3:** Primers used for quantitative real-time PCR (qPCR).

Calr-F	GGGAACCGCCAGTGATACAG
Calr-R	TTCTGGGTGGATCCAAGTGC
Calr-F	TGAAAACTTTGCTGTGCTGGG
Calr-R	CCCATGTCTCGTTGCCAAAC
Actb	CACGCCATCCTGCGTCTGGA
Actb	AGCACCGTGTTGGCGTAGAG

### Preparation of cell lysates and isolation of cell surface proteins

The cell lysates were obtained by solubilising cells in RIPA buffer (50 mM Tris-HCl, 150 mM NaCl, 0.1% SDS, 1% IGEPAL Ca-630, 0.5% sodium deoxycholate, pH 8.0) containing protease inhibitor cocktail (Sigma) for 30 min at room temperature (RT). The amounts of protein were determined with a bicinchoninic acid protein assay kit (Sigma). Isolation of cell surface proteins was performed using a Pierce® Cell Surface Protein Isolation Kit (Thermo Scientific) according to the manufacturer's instructions.

### Concanavalin A affinity chromatography

ConA affinity chromatography was performed as described by Soper et al. (Soper and Aird, 2007).

### SDS-PAGE and western blotting

The presence of porcine CRT in cell lysates was confirmed by Western blotting using primary rabbit monoclonal antibody directed against CRT (Cell Signaling, D3E6, XP® Rabbit mAb #12238) and horseradish peroxidase (HRP)-conjugated goat anti-rabbit immunoglobulins (DAKO, #00077731).

The binding of the FimH proteins to cell lysates was analyzed by far-Western blotting. Cell lysates or purified cell surface proteins separated by SDS-PAGE in 12% gel were transferred to nitrocellulose (Bio-Rad), blocked overnight with 3% BSA in TBST (TBS supplemented with 0.1% Triton X-100) and incubated with FimH proteins (0.1 mg/ml) for 4 h at RT. Immunodetection of FimH proteins was performed using primary rabbit polyclonal antibodies against FimH (Kisiela et al., 2005) and horseradish peroxidase (HRP)-conjugated goat anti-rabbit immunoglobulins (DAKO, #00077731).

### 2D electrophoresis

The samples (300 μg of total protein) were lysed in extraction buffer (7 M urea, 2 M thiourea, 4% w/v CHAPS, 2% Brij, 2% w/v 3-10 ampholytes, 40 mM Tris, and 100 mM DTT) and precipitated with ice-cold acetone for 2 h at −20°C. The first dimension was run using pH 3–7, 17 cm NL ReadyStrip™ IPG Strips (Bio-Rad). Prior to the second dimension SDS-PAGE, focused IPG strips were reduced with DTT (Sigma) in equilibration buffer (7 M urea, 2 M thiourea, 4% w/v CHAPS, 0.5% w/v 3–10 ampholytes, and 100 mM DTT) and alkylated with iodoacetamide (2.5% w/v). After equilibration, strips were placed on the top of 10% SDS polyacrylamide gels and held with molten 0.5% (w/v) agarose. After 2-DE separation, gels were either visualized with colloidal Coomassie brilliant blue G-250 according to the Hoving protocol (Westermeier, 2006) or transblotted onto nitrocellulose membranes. Selected spots were cut out from the gel and digested with trypsin using the All-in-One Trypsin Digestion Kit (Sigma).

### Protein identification by MALDI-TOF MS analysis of tryptic peptides

After tryptic digestion, all samples were acidified with formic acid (FA) and desalted on a 0.6 μl ZipTip C18 (Millipore) according to the manufacturer instructions. Peptides were eluted using 50% acetonitrile with 0.1% FA in water. Afterwards, 1 μl of each sample was mixed with 1 μl of saturated α-cyano-4-hydroxycinnamic acid (4-HCCA) matrix in 50% acetonitrile in water with 0.1% FA, and 1 μl of the sample/matrix solution was spotted onto the MALDI steel plate. Peptides were measured by matrix-assisted laser desorption/ionization-time of flight/time of flight mass spectrometry (MALDI-TOF-TOF-MS/MS) on an Ultraflex Extreme MALDI-TOF-TOF mass spectrometer (Bruker Daltonics, Bremen, Germany). The instrument was operated in positive ion reflection mode with an acceleration voltage of 25 kV. Spectra were acquired in the range of 700–3,500 Da with an average of 2,500 laser shots. Calibration of the MALDI-TOF mass spectrometer was performed with the peptide calibration standard (Bruker Daltonics, Bremen, Germany). All spectra were acquired by flexControl and then evaluated by flexAnalysis 3.4.7.76.0 software (Bruker Daltonics, Bremen, Germany).

Calibrated spectra were submitted to database searches using the MASCOT search engine (Matrix Science; http://www.matrixscience.com/). The search was performed with up to 1 trypsin miss-cleavages, carbamidomethylation of cysteine as a fixed modification, and oxidation of methionine as a potential modification. Peptide mass tolerance was set to ±0.45 Da, and MS/MS fragment ion tolerance was set to ±0.1 Da. A MASCOT search of all data was made against the SwissProt protein database restricted to mammalian taxonomy (66345 sequences).

### Recombinant proteins

Recombinant FimH adhesins were obtained as described in our previous paper (Grzymajlo et al., 2013) and named CFimH (active FimH variant from *S*. Choleraesuis), EFimH (active FimH variant from *S*. Enteritidis), and C63FimH (inactive FimH variant from *S*. Choleraesuis). Recombinant porcine calreticulin was obtained from CUSABIO (USA/China).

### Real-time interaction analysis by surface plasmon resonance

The binding of FimH adhesins to recombinant porcine CRT immobilized on CM5 sensor chips (GE Healthcare) was analyzed by SPR using a BIAcore T200. CRT immobilization level corresponds to approximately 150 RU. All binding experiments were carried out at 25°C with a 30 μl min^−1^ flow rate. To determine FimH affinity to CRT, 100 μl of five different concentrations (8, 4, 2, 1, and 0.5 μM) of FimH variant solutions, as well as a sample buffer blank, were passed over the ligand-immobilized chip surface (association phase) for 2 min, followed by 10 min of dissociation with running buffer. The same samples were passed over a control chip surface without immobilized ligand. Three replicates of each analyte concentration were injected. The resulting sensorgrams were obtained first by subtracting the buffer blank from curves recorded for the interactions of FimH proteins with CRT. Then, the curves recorded when adhesins were passed over the blank surface were subtracted from such sensorgrams. The equilibrium constants were determined using BIAevaluation 3.1 software. For global fitting, a 1:1 Langmuir binding model with an included mass transport step was applied based on criteria provided by the BIAevaluation handbook.

### Invasion and adhesion assay

For the invasion assay, *Salmonella*, after the fifth passage, were washed in PBS, resuspended in cell culture medium and adjusted by dilution to provide an MOI (multiplicity of infection) of 10:1 bacteria to host cells in culture wells of a 6-well plate. Confluent monolayers were infected for 1 h, followed by an additional hour of incubation with culture media containing 50 μg/ml of gentamicin (Sigma). After incubation, cells were washed three times with PBS and lysed with 0.1% Triton X-100 (Sigma) for 10 min. Bacterial suspensions were serially diluted with PBS, plated on LB-agar plates, and incubated overnight at 37°C, and the colonies were then counted to calculate the CFU.

For the adhesion assay, bacteria and cells were grown as described for the invasion assay. Infection was performed at an MOI of 50:1 bacteria to host cells. After 2 h of infection, cells were washed and plated as described for invasion assay. The percentage of dead cells was measured using bromophenol blue. Each assay was conducted in triplicate and was independently repeated at least three times.

### Protein and antibody blocking

Confluent monolayers of tested cells in a 96-well plate were incubated for 1 h with a monoclonal antibody against CRT (D3E6 XP®Rabbit mAb #12238), an antibody against CRT (Antibodies Online, Rabbit polyconal Ab #0805) that does not recognize porcine CRT at a 1:50 dilution, or with a FimH adhesin (CFimH or EFimH) with a final concentration of 0.1 mg/ml. Cells were incubated at 20°C for 1 h to allow for antibody or protein binding; then, SCΔ*fimH*/C bacteria (MOI 50:1) were added to quadruplicate wells, and the adhesion assay procedure was applied.

### Flow cytometry

Cells were grown to confluence in 6-well plates, detached with Accutase solution (Biowest), washed with staining buffer (PBS buffer supplemented with 1% bovine serum albumin and 0.002% NaN_3_) and incubated with or without 1 μl of calreticulin (D3E6) XP®Rabbit mAb #12238 for 2 h at 4°C. After the incubation, the cells were washed with staining buffer and incubated with 0.125 μg of fluorochrome-conjugated secondary antibody, PE Donkey anti-rabbit IgG (minimal x-reactivity) antibody (Biologend, clone Poly4064) or with 0.125 μg Alexa Fluor®647 Donkey anti-rabbit IgG (minimal x-reactivity) antibody (Biologend, Clone Poly4064), for 30 min at 4°C. Afterwards, cells were washed twice with staining buffer and fixed in 1% buffered formaldehyde. The samples were then immediately analyzed with a FACSCalibur II cytometer (BD Pharmingen). All analysis was carried out with Weasel 3.0.2 software (http://en.bio-soft.net/other/WEASEL.html).

### Immunofluorescence and microscopy analysis

Cells (5 × 10^3^) were grown on 18 mm glass coverslips for 24 h in a humidified cell culture chamber (37°C, 5% CO_2_) and subsequently fixed in 2% paraformaldehyde at RT for 15 min. Afterwards, the cells were briefly rinsed in PBS and permeabilized with PBS containing 0.1% Triton X-100 for 5 min at 4°C. Permeabilization solution was next aspirated, and cells were blocked with FCS (20 min, RT) and further incubated overnight with primary anti-calreticulin antibody (D3E6; dilution 1:50) at 4°C. Cells were rinsed with PBS and incubated for 45 min at RT with the Alexa Fluor®633 anti-rabbit secondary antibody (Thermo Fischer Scientific; dilution 1:400) in the dark, and nuclei were stained with mounting medium containing DAPI (Abcam, UK). Images were acquired on an LSM 510 META microscope (Carl Zeiss, GmbH, Germany) using a PLAN-APOCHROMAT 63x/1.4OIL DICM27 objective. Image acquisition was performed using ZEN 2009 Light Edition software.

### Statistical analysis

All statistical calculations were performed in GraphPad (GraphPad Software, Inc., La Jolla, CA, USA). The shape of the data distribution was assessed with the Shapiro normality test and analysis of quantile-normal plots. Mann-Whitney test (nonparametric), Student's *t*-test (parametric) or one-way ANOVA with *post hoc* Dunn's multiple comparison test were performed based on the shape of data distribution. All data collected in this study were obtained from at least three independent experiments for each condition. A *p*-value of less than 0.05 was considered statistically significant. Data are presented as the means ± standard deviation (SD).

## Results

### Identification of calreticulin as a receptor for *Salmonella* choleraesuis FimH

**Figure 1:**
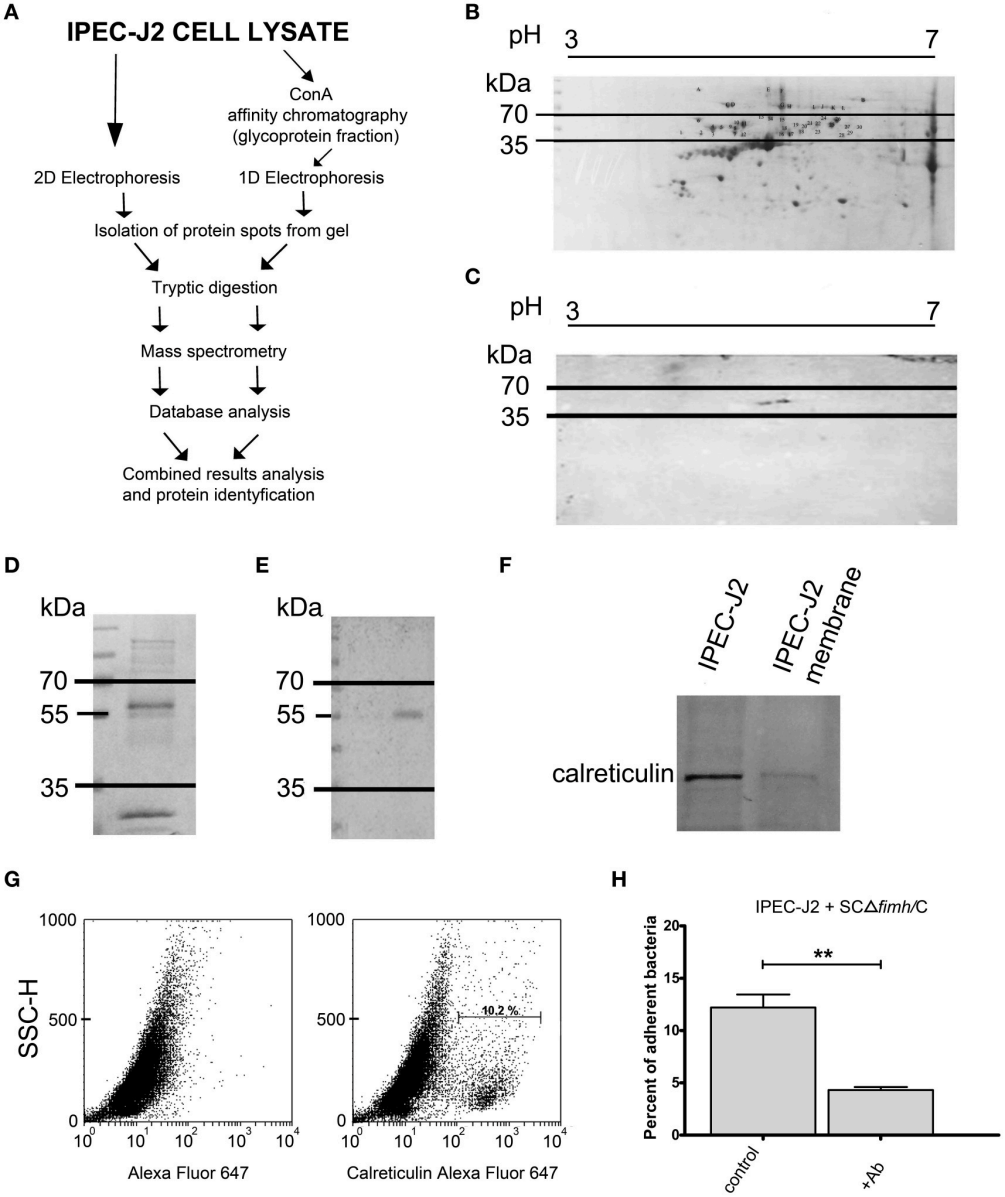
Identification of IPEC-J2 receptor for *Salmonella* Choleraesuis FimH adhesion. **(A)** Scheme of the receptor identification procedures. **(B)** 2D electrophoresis of the IPEC-J2 cell lysate. IPEC-J2 cells were lysed in extraction buffer and stained with Coomassie brilliant blue. The horizontal axis shows the isoelectric point, and the vertical axis shows molecular weight (kDa). The molecular mass range of proteins cut from the gel is marked by black lines. **(C)** Far-Western blotting analysis of CFimH binding to proteins from the IPEC-J2 cell lysate. Cell lysates, equivalent to 300 μg of protein, were separated in two dimensions (pH and molecular weight) and transferred onto nitrocellulose. The horizontal axis shows the isoelectric point, and the vertical axis shows molecular weight (kDa). **(D)** Coomassie-stained 1D electrophoresis of IPEC-J2 glycoprotein fraction (20 μg of protein per line) obtained by ConA affinity chromatography. The migration of protein standards (in kDa) is indicated on the left. **(E)** Far-Western blotting analysis of CFimH binding to the ConA fraction of the IPEC-J2 cell line. Cell lysates, equivalent to 20 μg of protein, were separated by SDS–PAGE and transferred onto nitrocellulose. The migration of protein standards (in kDa) is indicated on the left. **(F)** Detection of calreticulin by Western blotting with anti-calreticulin rabbit monoclonal antibodies in IPEC-J2 whole cell lysate and IPEC-J2 membrane fraction. Cell lysates, equivalent to 20 μg of protein, were separated by SDS–PAGE and transferred onto nitrocellulose. **(G)** Flow cytometry analysis of IPEC-J2 cells. The cells were detached with Accutase, washed with PBS, incubated with rabbit anti-calreticulin antibody followed by PE-conjugated secondary antibody, fixed with 1% buffered formaldehyde and immediately analyzed on a cytometer. The percentage of CRT-positive cells is presented on the image. **(H)** Adherence of SCΔ*fimH*/C to IPEC-J2 cells preincubated for 1 h with PBS buffer (control) or anti-calreticulin monoclonal antibody (Ab). Data are means ± SD in triplicate assays, which were independently repeated three times. Here and in all other assays ^*^p < 0.05, ^**^p < 0.01, ^***^p < 0.001.

To identify the host receptor for FimH protein, we applied two independent pathways (Figure 1A). First, IPEC-J2 cell lysates were subjected to 2D electrophoresis and either stained with Coomassie brilliant blue (Figure 1B) or transferred onto a nitrocellulose membrane. The presence of protein bound by *S*. Choleraesuis FimH was analyzed by far-Western blotting using recombinant *S*. Choleraesuis FimH (CFimH) protein. As CFimH adhesin bound to a protein spot with a molecular mass approximately 55 kDa and pI in a range between 4 and 6 (Figure 1C), all spots from the Coomassie-stained 2D gel with molecular masses between 35 and 70 kD and pI between 4 and 6 were cut out, digested with trypsin and analyzed by MALDI-MS and peptide mass fingerprinting.

Alternatively, IPEC-J2 cell lysates were subjected to affinity chromatography on Concanavalin A (ConA), and the fraction enriched in ConA-binding glycoproteins was subjected to SDS-PAGE and far-Western blotting with CFimH (Figures 1D,E). In agreement with the results presented above, a protein band with a molecular mass approximately 55 kDa was detected by CFimH adhesin; thus, protein bands of apparent molecular mass between 35 and 70 kDa were cut from the gel, digested with trypsin and analyzed by MALDI-MS and peptide mass fingerprinting. The results from both pathways were combined, and the analysis has provided a probability-based Mowse score for the best matching protein hits, and using the MASCOT analysis tool, the receptor has been identified as calreticulin.

To confirm MS data and show that IPEC-J2 cells express calreticulin, we analyzed cell lysate and the membrane fraction of IPEC-J2 cells by Western blotting with an anti-calreticulin antibody (Figure 1F). In addition, flow cytometry analysis revealed that CRT is expressed on the surface of IPEC-J2 cells (Figure 1G). To further prove that CRT is a receptor for *S*. Choleraesuis FimH adhesin, we also tested whether preincubation of IPEC-J2 cells with anti-calreticulin antibody could inhibit adhesion of *S*. Choleraesuis to porcine cells. It was found that anti-calreticulin antibody significantly (*p* < 0.01) inhibited binding of the SCΔ*fimH*/C *Salmonella* strain to IPEC-J2 cells (Figure 1H, Table 1).

### Porcine calreticulin is a receptor for host-adapted *Salmonella* choleraesuis FimH and not for broad host range *Salmonella* enteritidis FimH

**Figure 2:**
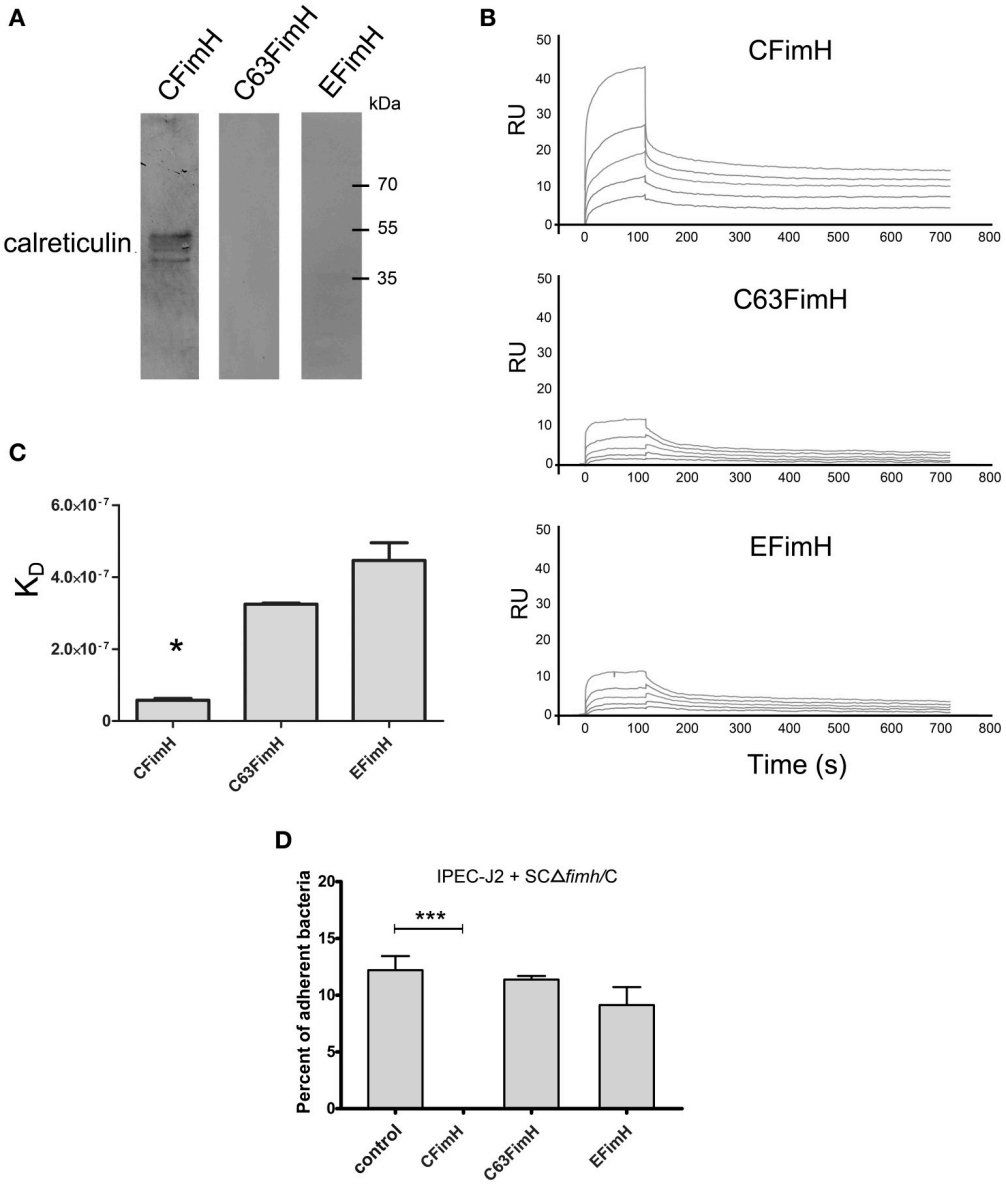
Interaction of FimH adhesin with porcine calreticulin. **(A)** Far-Western blotting analysis of FimH adhesin binding to recombinant porcine CRT. CRT (0.5 μg) was subjected to SDS–PAGE and transferred onto nitrocellulose. CFimH, C63FimH and EFimH were incubated with CRT immobilized on the membrane and then detected with anti-FimH rabbit polyclonal antibody and secondary anti-rabbit antibody. **(B)** Interaction of CFimH, C63FimH and EFimH with immobilized CRT analyzed with an SPR. Lines represent different concentrations of analyte (0.5, 1, 2, 4, and 8 μM) in HBS buffer. Binding data were collected at a flow rate of 30 μl/min. **(C)** Dissociation constants of scFimH adhesins determined by SPR analysis. **(D)** Adherence of SCΔ*fimH*/C to IPEC-J2 cells preincubated for 1 h with PBS buffer (control), CFimH adhesin, C63FimH adhesin or EFimH adhesin. Data are means ± SD in triplicate assays, which were independently repeated three times.

To show that calreticulin is a specific receptor for *S*. Choleraesuis FimH adhesin, we studied its interaction with variants of FimH proteins by far-Western blotting and surface plasmon resonance (SPR). It was found that, in static conditions, only active *S*. Choleraesuis FimH adhesin (CFimH) was able to bind to recombinant porcine CRT in contrast to inactive FimH (C63FimH) from *S*. Choleraesuis and active *S*. Enteritidis FimH (EFimH) (Figure 2A). It is worth to mention, that we observed three bands in far-western blot interaction between CFimH and CRT, probably because of different glycoforms of the recombinant CRT (see Figure S5). These data were further confirmed by direct kinetic analysis using SPR. For each allelic variant, analysis was carried out at different concentrations of recombinant FimH proteins (0.5, 1, 2, 4, and 8 μM; Figure 2B). According to SPR data, CFimH bound significantly stronger (KD = 6 × 10^−8^) to CRT in comparison with that of C63FimH and EFimH (K_*D*_ = 2 × 10^−7^) (Figure 2C).

To verify this interaction on the cell line model, we tested whether the purified FimH could block the *Salmonella*-IPEC-J2 cell interaction and lead to decreased bacterial attachment. Cells were plated and incubated with CFimH or EFimH and then challenged with our standard adhesion test with SCΔ*fimH*/C strain (Figure 2D). It was found that only active CFimH strongly inhibited the interaction between bacteria and tested cells (*p* < 0.001), indicating that porcine calreticulin is a specific receptor for *S*. Choleraesuis FimH adhesin.

**Figure 3:**
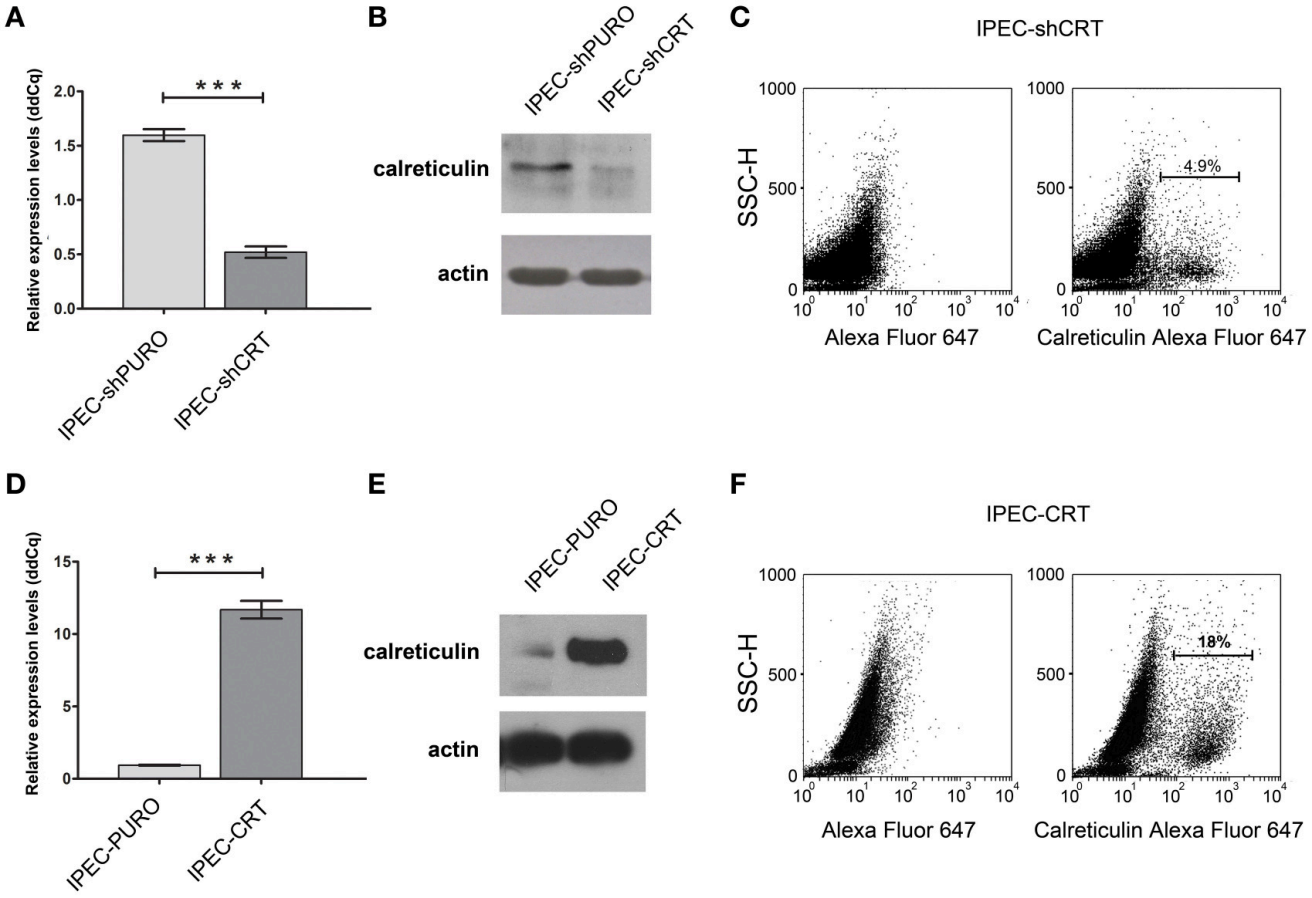
Characteristics of IPEC-J2 cells with the calreticulin gene silenced or overexpressed. **(A)** Expression of calreticulin mRNA in control IPEC-shPURO cells and IPEC-shCRT with decreased expression of calreticulin. qPCR was used to analyse expression of mRNAs. The expression levels were normalized against the *Actb* gene. **(B)** Western blotting analysis of CRT expression with anti-calreticulin rabbit monoclonal antibodies in IPEC-shPURO cells and IPEC-shCRT cells with decreased expression of CRT. Cell lysates, equivalent to 20 μg of protein, were separated by SDS–PAGE and transferred onto nitrocellulose. **(C)** Flow cytometry analysis of IPEC-shCRT cells. The cells were detached with Accutase, washed with PBS, incubated with rabbit anti-calreticulin antibody followed by Alexa Fluor 647-conjugated secondary antibody, fixed with 1% buffered formaldehyde and immediately analyzed with a cytometer. The percentage of CRT-positive cells is presented on the image. **(D)** Expression of calreticulin mRNA in IPEC-PURO cells and IPEC-CRT cells with overexpression of calreticulin. qPCR was used to analyse the expression of mRNAs normalized against the *Actb* gene. **(E)** Western blotting analysis of CRT expression with anti-calreticulin rabbit monoclonal antibodies in IPEC-PURO cells and IPEC-CRT cells with overexpression of calreticulin. Cell lysates, equivalent to 20 μg of protein, were separated by SDS–PAGE and transferred onto nitrocellulose. **(F)** Flow cytometry analysis of IPEC-CRT cells. The cells were detached with Accutase, washed with PBS, incubated with rabbit anti-calreticulin antibody followed by PE-conjugated secondary antibody, fixed with 1% buffered formaldehyde and immediately analyzed with a cytometer. The percentage of calreticulin-positive cells is presented on the image.

To support our hypothesis that porcine calreticulin is a specific receptor for an allelic variant of FimH adhesin from *S*. Choleraesuis, the adhesion and invasion of *S*. Choleraesuis isogenic strains expressing different variants of FimH adhesin (Table 1, 2) to porcine IPEC-J2 cells with different CRT expression levels were studied. To generate a specific loss-of-function phenotype, IPEC-J2 cells expressing CRT were transduced with the pLVTHM/shCRT construct to inhibit expression of the receptor. Such cells, named IPEC-shCRT, were characterized by a strongly decreased level of calreticulin mRNA (Figure 3A) and highly decreased binding of anti-calreticulin antibodies to cell lysates in comparison to control IPEC-shPURO cells obtained after transduction of IPEC-J2 cells with the pLVTHM/PURO vector (Figure 3B). To create a gain-of-function cellular model, we transduced IPEC-J2 cells with the pRRL-PURO-CRT-IRES expression vector, and the resulting cell population, which overexpressed calreticulin at the mRNA and protein levels (Figures 3D,E), was named IPEC-CRT. Control IPEC-PURO cells with basal CRT expression level were obtained by transduction of IPEC-J2 cells with the pRRL-PURO-IRES-vector. The membrane level of CRT was decreased in the silencing model and upregulated in the overexpression model as presented by flow cytometry plots (Figures 3C,F). The cells growth rate do not change after genetic modifications (Figure S2).

**Figure 4:**
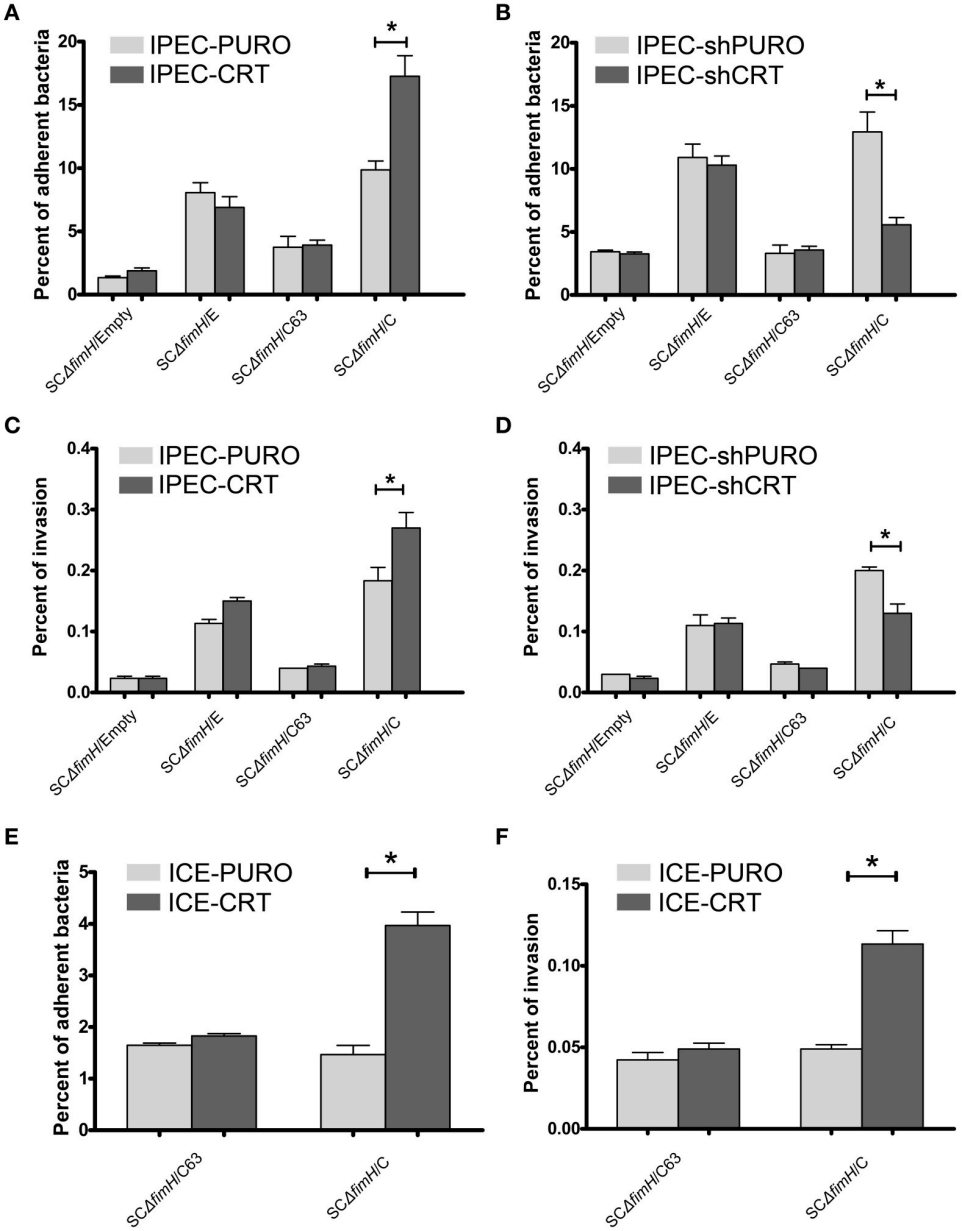
Adherence and invasion of *S*. Choleraesuis isogenic mutant strains to enterocytes with overexpression (IPEC-CRT cells), suppressed expression (IPEC-shCRT cells) or neoexpression (ICE-CRT) of calreticulin. **(A)** Adhesion assay with IPEC-PURO cells and IPEC-CRT cells. **(B)** Adhesion assay with IPEC-shPURO cells and IPEC-shCRT cells. **(C)** Invasion assay with IPEC-PURO and IPEC-CRT cells. **(D)** Invasion assay with IPEC-shPURO and IPEC-shCRT cells. **(E)** Adhesion assay with ICE-PURO cells and ICE-CRT cells. **(F)** Invasion assay with ICE-PURO and ICE-CRT cells. Data are means ± SD in triplicate assays, which were independently repeated three times.

To investigate how the calreticulin expression level affects bacterial attachment and invasion, we used our gain-of-function (IPEC-CRT) and loss-of-function (IPEC-shCRT) cell models. Isogenic strains of *S*. Choleraesuis that express non-binding (SCΔ*fimH/*C63) and high-binding (SCΔ*fimH*/C) FimH variants from *S*. Choleraesuis and FimH variant from *S*. Enteritidis (SCΔ*fimH*/E), as well as *S*. Choleraesuis without FimH expression (SCΔ*fimH*/Empty), were compared. This experiment revealed that only SCΔ*fimH*/C binds significantly better (*p* < 0.01) to IPEC-CRT cells (Figure 4A). Thus, under these conditions, *Salmonella* expressing active FimH from *S*. Choleraesuis is able to adhere better thanks to a higher amount of CRT expressed on the cell surface. When we compared adhesion of all strains to IPEC-shCRT cells representing model with decreased CRT expression (Figure 4B), the pattern was generally the same. Together with lower expression of calreticulin, the SCΔ*fimH*/C strain binds to IPEC-shCRT cells significantly weaker (*p* < 0.05) than to control cells. Taking these two models into account, we found that the expression level of calreticulin correlates with the level of adhered SCΔ*fimH*/C, which indicates that the receptor is involved in mediating infection specifically for this variant of FimH. When bacterial internalization was examined (Figures 4C,D), again only the isogenic strain SCΔ*fimH*/C invaded IPEC-CRT cells significantly better (*p* < 0.01) and IPEC-shCRT significantly weaker (*p* < 0.05) than that of control IPEC cells. However, the invasion level was low and therefore its biological significance still needs to be proven. As expected, strains producing FimH variants that are able to bind to oligomannosidic structures (SCΔ*fimH*/C, SCΔ*fimH*/E) bind to both epithelial cell variants up to 8-fold higher than that of strains with inactive FimH and up to 20-times higher than that of the FimH knockout strain.

We next wanted to evaluate whether the role of calreticulin as a FimH-dependent host-specific receptor was restricted to IPEC-J2 cells by testing epithelial intestinal cells from other species. Transduction of ICE-1 cells of mouse origin, with the pRRL-PURO-CRT-IRES expression vector results in strong neoexpression of porcine calreticulin on protein level (Figure S3). When we perform adhesion and invasion tests using mouse intestinal epithelial cells with neoexpression of porcine calreticulin (ICE-CRT) with two isogenic strains, SCΔ*fimH/*C63 and SCΔ*fimH*/C, profound differences were found in their binding patterns. Strain SCΔ*fimH*/C binds and invades ICE-CRT cells with porcine calreticulin expression significantly better (*p* < 0.05) than those of control ICE-PURO cells (Figures 4E,F). Importantly, binding of *S*. Choleraesuis isogenic variants to cells of mouse origin was significantly weaker (*p* < 0.05) when compared with binding to porcine cells, again highlighting the host specificity of such an interaction. Taken together, the presence of porcine calreticulin correlates with adhesive and invasive properties of *S*. Choleraesuis with active FimH-variant, which supports the host specificity hypothesis of this interaction.

### Impact of infection of *S*. Choleraesuis with active FimH adhesin on calreticulin membrane level in IPEC-J2 cells

**Figure 5:**
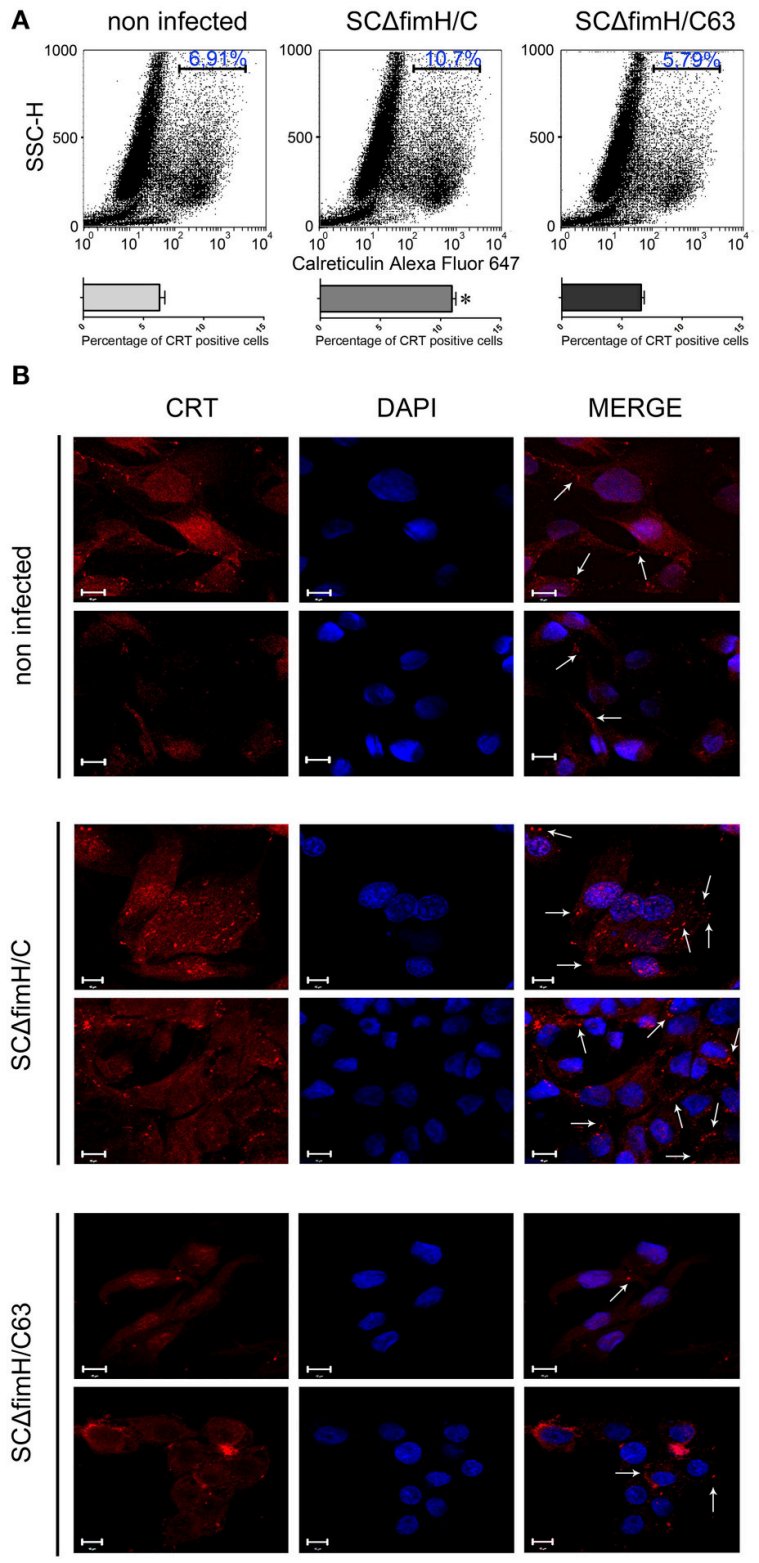
Infection of IPEC-J2 cells with *S*. Choleraesuis with active and inactive FimH variants. **(A)** Flow cytometry analysis of IPEC-CRT cells 4 h after *S*. Choleraesuis infection. The cells were detached with Accutase, washed with PBS, incubated with rabbit anti-calreticulin antibody followed by PE-conjugated secondary antibody, fixed with 1% buffered formaldehyde and immediately analyzed with a cytometer. The percentage of calreticulin-positive cells is presented on the image. **(B)** Localization and distribution of CRT in IPEC-J2 cells infected with *S*. Choleraesuis. Images were acquired on an LSM 510 META microscope (Carl Zeiss, GmbH Germany) using a PLAN-APOCHROMAT 63x/1.4 OIL DIC M27 objective. Image acquisition was performed using ZEN 2009 Light Edition software. Bars represent 10 μm. Membrane CRT organized in dot aggregates are indicated by arrows.

When we investigated the membrane CRT level 4 h after *Salmonella* infection using flow cytometry, noticeable differences were found between SCΔ*fimH*/C63 and SCΔ*fimH*/C infected cells. In cells infected with the active strain, a significantly higher number of cells expressing surface calreticulin was observed when compared to the number of inactive strain infected or uninfected cells (Figure 5A). Interesting differences in CRT localisation between SCΔ*fimH*/C63 and SCΔ*fimH*/C infected cells were found using confocal microscopy. When compared to uninfected cells as well as to cells infected with inactive strain, IPEC-J2 cells infected with SCΔ*fimH*/C present higher surface expression of calreticulin. Furthermore, surface CRT on such cells was organized in dot aggregates (Figure 5B and Figure S4), which can be a potential entry site for pathogens. Taken together, these results suggest that there is an association between infection of *Salmonella* Choleraesuis with active FimH and surface calreticulin presence and organization.

## Discussion

Host specificity of bacterial pathogens, including *Salmonella enterica*, is currently one of the most intensively studied problems in modern microbiology. The majority of studies are focused on this phenomenon only from pathogen side. Nevertheless, host-pathogen interactions as well as adaptation to a specific host is dependent on not only the pathogen but also host factors. *Salmonella enterica* as a species is a great example of closely related bacteria with different host specificities (Baumler and Fang, 2013; Yue et al., 2015) with clear division between generalist serovars able to infect a broad range of hosts and specialists, which infect from one up to several host species. Such serovars, like *S*. Enteritidis or *S*. Typhimurium, usually infect many different animal species and therefore are called broad host-range. In contrast, infections caused by host-restricted *Salmonella* (e.g., *S*. Gallinarum, *S*. Typhi) lead to severe systemic disease, which is described as typhoid fever. There are several molecular mechanisms, including bacterial adherence, replication in host cells, species-specific toxins or metal acquisition involved in host specificity of *Salmonella* (reviewed in Baumler and Fang, 2013). It was previously shown that FimH adhesin single nucleotide polymorphisms (SNPs) impact adhesion properties of *E. coli* (Sokurenko et al., 1994) and *Salmonella* (Boddicker et al., 2002; Grzymajlo et al., 2010), as well as *Salmonella* host specificity (Boddicker et al., 2002; Grzymajlo et al., 2010, 2013; Kisiela et al., 2012; Yue et al., 2015). Furthermore, host-adapted *S*. Choleraesuis was previously indicated as a great example of host-specific adhesion (Yue et al., 2015). Our former studies revealed that a membrane protein found in the porcine IPEC-J2 cell line was specifically recognized by FimH adhesin from *S*. Choleraesuis and not by FimH from *S*. Enteritidis (Grzymajlo et al., 2013). Therefore, the goal of this study was to identify a host-specific receptor for FimH adhesin expressed by porcine enterocytes and present FimH adhesin from *S*. Choleraesuis involvement in host-specific ligand recognition.

Adhesion to host tissues involves a specific interaction between pathogen and host proteins expressed at the surface of the cells. Here, we focus on type 1 fimbriae and its adhesin FimH, one of the most abundant *Enterobacteriaceae* adhesin structures. A well characterized FimH adhesin from *E. coli* mediate adhesion to number of glycosylated and non-glycosylated host receptors, including collagens, laminin, fibronectin, clathrin, integrins, and many others (Sokurenko et al., 1997; Ashkar et al., 2008; Eto et al., 2008; Mossman et al., 2008). Despite *Salmonella*'s FimH is also one of the most extensively studied microbial adhesins, there is only limited information available regarding its host receptors (Leusch et al., 1991; Ghosh et al., 1996; Kukkonen et al., 1998; Pier et al., 1998; Lyczak and Pier, 2002) with only one well-described host receptor to date: glycoprotein-2 expressed on M-cells (Hase et al., 2009). Since M-cells are a known site for pathogen entry, up to 95% of small intestine cells are enterocytes. Therefore, it would be advisable to search new receptors for bacterial adhesion and internalization in these cells.

Interaction of *Salmonella* FimH adhesin and host cells has been extensively studied and proven on various cell lines models, including Hela, Hep-2 or HT-29 and Caco-2 cells (Hancox et al., 1997; Boddicker et al., 2002; Kisiela et al., 2006). The IPEC-J2 cell line used in our study was previously used in a host-pathogen interaction study (Brosnahan and Brown, 2012), including *Salmonella* species interaction (Schierack et al., 2006). Unlike cell lines derived from tumors (e.g., HT-29, Caco-2), IPEC-J2 is a non-transformed epithelial cell line isolated from piglet mid-jejunum. It can form polarized monolayer epithelial cells and serve as a great model for microbiological experiments of host–pathogen intestinal interactions by supporting interactions with a variety of bacterial species. In our previous study, we have shown that FimH adhesin from *S*. Choleraesuis interacts directly with a specific protein band from IPEC-J2 lysate (Grzymajlo et al., 2013), and here, we identify this binding partner as calreticulin. This study represents the first report regarding a role of calreticulin as a host-specific receptor for *S. enterica* serovar Choleraesuis FimH adhesin and also the first proof of the activity of mammalian calreticulin as a bacterial receptor.

Calreticulin is a well-known ER chaperon protein responsible for proper folding of glycoproteins as well as regulation of calcium metabolism (Gold et al., 2010). Currently, it is known that CRT can be localized in intracellular and extracellular compartments. Moreover, despite no clear mechanism describing how calreticulin is transported to the cell membrane being known to date, there is plenty of evidence regarding the presence of CRT on the cell surface (Jeffery et al., 2011; Wiersma et al., 2015). Among a number of different functions of CRT outside the ER, such as cell adhesion (Nanney et al., 2008), cellular proliferation and phagocytosis of apoptotic cells (Gardai et al., 2005), calreticulin was shown to act as a cellular receptor for C1q, mannose-binding lectins and focolins (Lacroix et al., 2009), and most recently, also as a microbial binding molecule with a phagocytosis-enhancing capacity (Liu et al., 2013).

Here, we present a number of pieces of evidence that calreticulin not only is present at the surface of the IPEC-J2 cell line (Figure 1) but can also act as a host-specific receptor for *S*. Choleraesuis FimH adhesin. First, we observed a direct binding interaction between porcine calreticulin and the active variant of FimH adhesin from *S*. Choleraesuis using purified proteins in static and dynamic conditions (Figure 2). Moreover, this interaction did not occur with FimH adhesin from *S*. Enteritidis since its binding constant to recombinant CRT is comparable to the binding of the inactive variant of FimH from *S*. Choleraesuis. The CRT-FimH interaction can be interrupted in the cell model by an anti-calreticulin antibody (Figure 1H), resulting in a significant decrease in binding of bacteria to the cell surface. Furthermore, purified *S*. Choleraesuis FimH adhesin can also inhibit cell-bacteria interaction as opposed to *S*. Enteritidis FimH adhesin (Figure 2). Thus, our results support the importance of host-specific receptor-ligand interactions.

It is widely accepted that type 1 fimbrial FimH adhesins bind to oligomannosidic structures, and we have shown that FimH proteins from *S*. Choleraesuis and *S*. Enteritidis are active in mannose-sensitive manner (Grzymajlo et al., 2013), whereas the V63G mutant (C63FimH) is mannose insensitive. We have shown that porcine CRT is able to bind ConA, and therefore, it can carry glycan chains. Moreover, binding of FimH to CRT is abolished after deglycosylation, which confirms that a specific protein-sugar conformation is important for the presented interaction as well as for *S*. Choleraesuis infection. Those findings support our earlier hypothesis (Grzymajlo et al., 2013) that the FimH-host receptor interaction depends on the proper presentation of high mannose structures on specific protein carriers.

To answer the question as to whether calreticulin can act as a host-specific receptor for FimH adhesin of *S*. Choleraesuis, we tested binding and internalization of bacterial mutants to IPEC-J2 cell lines with overexpression and shRNA knockdown of calreticulin. Our earlier studies revealed that single point mutations in FimH adhesins from *S*. Choleraesuis and *S*. Enteritidis are responsible for different binding pattern (Grzymajlo et al., 2013; Table 2). Thus, we constructed isogenic model of *S*. Choleraesuis with expression of different FimH variants. We have shown that only *S*. Choleraesuis with expression of the active FimH variant (SCΔ*fimH*/C) binds in significantly higher numbers to IPEC-J2 cells with overexpression of calreticulin (Figure 4). Higher expression of CRT on the surface of IPEC-CRT cells does not significantly impact binding of all the other tested strains. Similar results were obtained in the model with reduced expression of calreticulin. Only SCΔ*fimH*/C binds in a significantly lower number to IPEC-shCRT in comparison to wild-type cells. These data suggest that such an interaction is relevant in a physiological context. Our findings support former results (Kisiela et al., 2012; Grzymajlo et al., 2013; Yue et al., 2015) that allelic variants of FimH adhesin have an impact on host specificity by allowing recognition of a host receptor in a species-dependent manner. When the ectopic expression of porcine calreticulin in mice intestinal epithelial cells (ICE-1) was performed, the *S*. Choleraesuis binding pattern supports our host specificity receptor hypothesis. When we compared isogenic *Salmonella* strains expressing active and inactive *S*. Choleraesuis FimH, only the active variant adheres significantly stronger to ICE-1 cells expressing porcine CRT. Worth mentioning is that even the active strain adheres to wild-type mouse cells significantly weaker than to porcine cells, which again support the hypothesis of host specificity in a FimH-dependent manner (Yue et al., 2015).

Previous research has shown that *S*. Typhimurium is able to modify host cells to facilitate translocation through intestinal mucosa (Tahoun et al., 2012). In our study, *S*. Choleraesuis invasion was associated with changes in membrane CRT level in IPEC-J2 cells when compared to the receptor amount observed during invasion of *S*. Choleraesuis with the inactive FimH variant (Figure 5A). Moreover, we observed changes in CRT organization in cell membrane (Figure 5B). The interesting aspect is again that this process is strongly connected with the FimH variant, since from tested FimH adhesins, only active FimH from *S*. Choleraesuis induces calreticulin relocation. When we connect this with host specificity of *Salmonella* serovars, we can speculate that the increase of the CRT level in the membrane of enterocytes is connected with the higher invasion rate of *S*. Choleraesuis to its porcine host. Another question is how CRT can transduce the signal as well as take part in bacterial internalization. There is no clear evidence of cell surface calreticulin-direct signaling, but its surface exposure can be regulated, for example, by soluble factors, such as IL-8 (Sukkurwala et al., 2014), or by intracellular signal transduction through LRP1 (Garg et al., 2012).

In conclusion, we have identified a specific receptor for *S*. Choleraesuis type 1 fimbriae FimH adhesin expressed in swine intestinal cells as calreticulin. Moreover, it seems that the specific calreticulin- *S*. Choleraesuis FimH interaction plays an important role in *Salmonella* host specificity. To date, calreticulin has mainly been known as a chaperone protein present in the endoplasmic reticulum; however, recent studies have shown its contribution in a variety of cellular processes, including microbial binding and phagocytosis (Liu et al., 2013). Here, we confirm membrane localization of porcine calreticulin as well as its function in adhesion and internalization processes of *S*. Choleraesuis through FimH adhesin. Furthermore, latter finding is supported by the observation that *S*. Choleraesuis infection induces translocation of calreticulin to cell membrane. All of the aforementioned results lead to the general conclusion that *Salmonella* host specificity requires not only special mechanisms and proteins expressed by the pathogen but also specifically recognized receptors expressed by a specific host.

## Author contributions

KG: conceptualization, methodology, investigation, resources, writing original draft, visualization, funding acquisition; MU: writing-review and editing; JS: investigation; AK: investigation, writing-review and editing: RK: investigation, writing-review and editing; AJ: investigation; AB: investigation; PS: writing-review and editing.

### Conflict of interest statement

The authors declare that the research was conducted in the absence of any commercial or financial relationships that could be construed as a potential conflict of interest.

## Supplementary material

Figure S1 Binding of *Salmonella* Choleraesuis mutants to anti-FimH antibody and to RNaseB as a glycoprotein standard.

Figure S2 Proliferation assay. Cell proliferation was determined using the SRB assay as described in the Materials and Methods. The values are shown as the means ± SD from two independent assays with 10 repetitions in each assay.

Figure S3 Detection of calreticulin by Western blotting. Cell lysates, equivalent to 20 μg of protein, were separated by SDS–PAGE and transferred onto nitrocellulose. **(A)** In ICE-PURO and ICE-CRT whole cell lysate with anti-calreticulin rabbit monoclonal antibodies-(D3E6) XP® Rabbit mAb #12238 (Cell signaling). **(B)** In ICE-PURO and ICE-CRT whole cell lysate with anti-calreticulin rabbit polyclonal antibody (ABIN361835) (Antibodies Online) which do not recognize porcine calreticulin.

Figure S4 Localization and distribution of CRT in IPEC-J2 cells. Images were acquired on an LSM 510 META microscope (Carl Zeiss, GmbH Germany) using a PLAN-APOCHROMAT 63x/1.4 OIL DIC M27 objective. Image acquisition was performed using ZEN 2009 Light Edition software. Bars represent 10 μm. Membrane CRT organized in dot aggregates are indicated by arrows.

Figure S5 Interaction with recombinant porcine calreticulin. **(A)** Far-Western blotting analysis of FimH adhesin binding to recombinant porcine CRT. CRT (0.5 μg) was subjected to SDS–PAGE and transferred onto nitrocellulose. CFimH, C63FimH and EFimH were incubated with CRT immobilized on the membrane and then detected with anti-FimH rabbit polyclonal antibody and secondary anti-rabbit antibody. **(B)** Detection of recombinant calreticulin (0.5 μg) by Western blotting with anti-calreticulin rabbit monoclonal antibodies secondary anti-rabbit antibody. Protein was separated by SDS–PAGE and transferred onto nitrocellulose.
