# Supplementary figures and images for: The Novel Type 1 Fimbriae FimH Receptor Calreticulin Plays a Role in Salmonella Host Specificity
Source: Front Cell Infect Microbiol. 2017 Jul 19;7:326. doi: 10.3389/fcimb.2017.00326 (PMC5516122; doi:10.3389/fcimb.2017.00326)

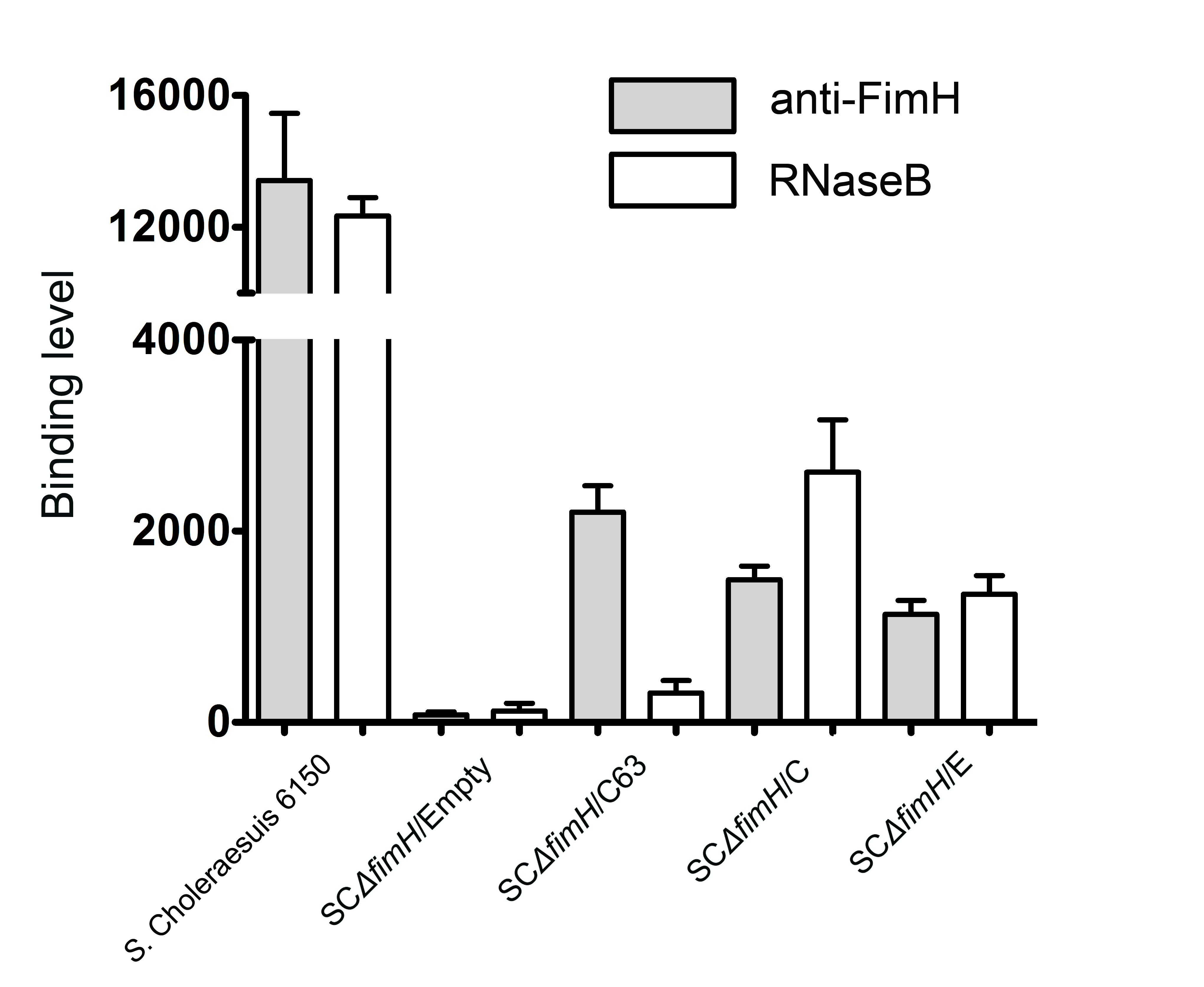

Supplement: Figure S1 — Binding of Salmonella Choleraesuis mutants to anti-FimH antibody and to RNaseB as a glycoprotein standard. [file Image1.JPEG]

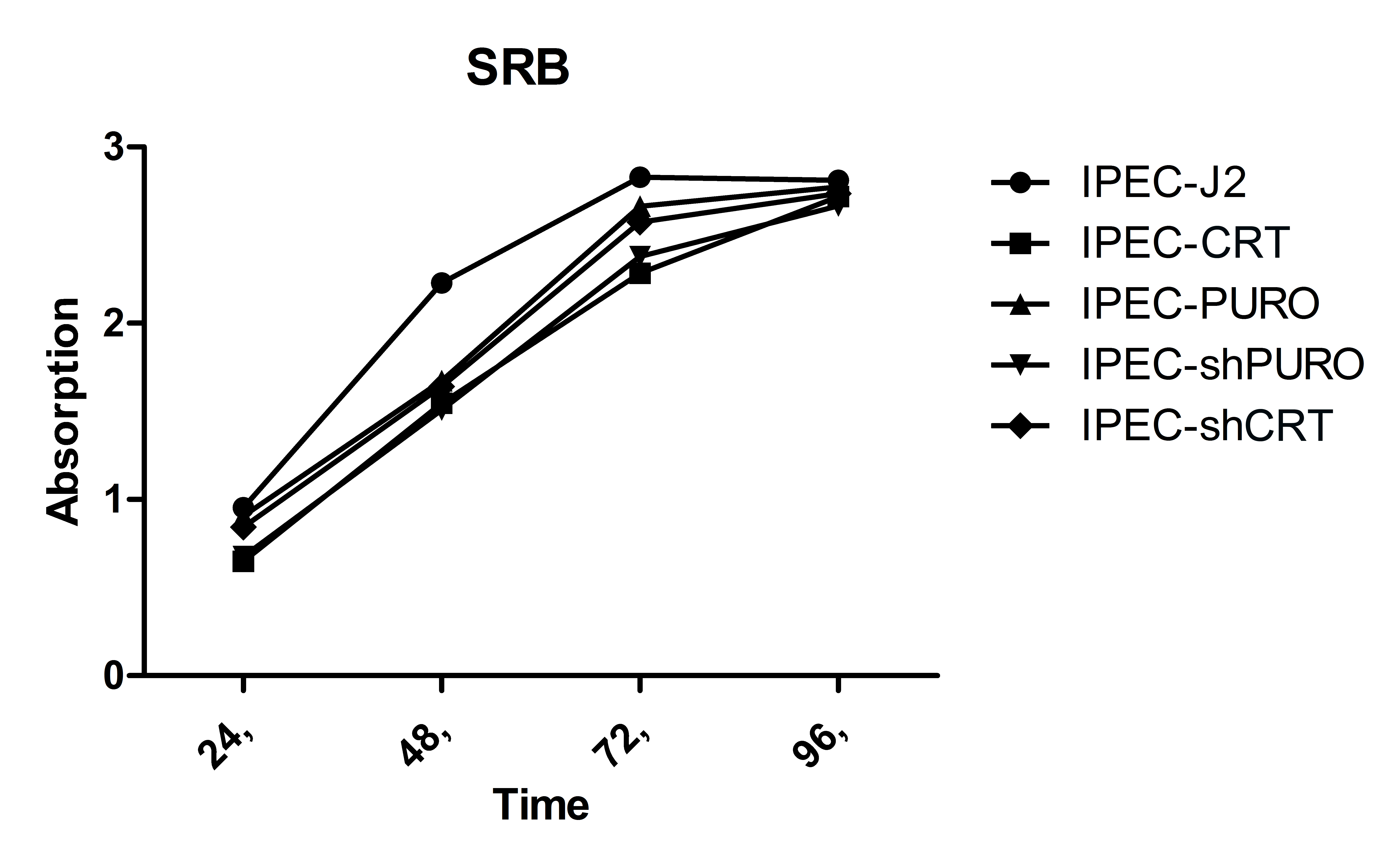

Supplement: Figure S2 — Proliferation assay. Cell proliferation was determined using the SRB assay as described in the Materials and Methods. The values are shown as the means ± SD from two independent assays with 10 repetitions in each assay. [file Image2.JPEG]

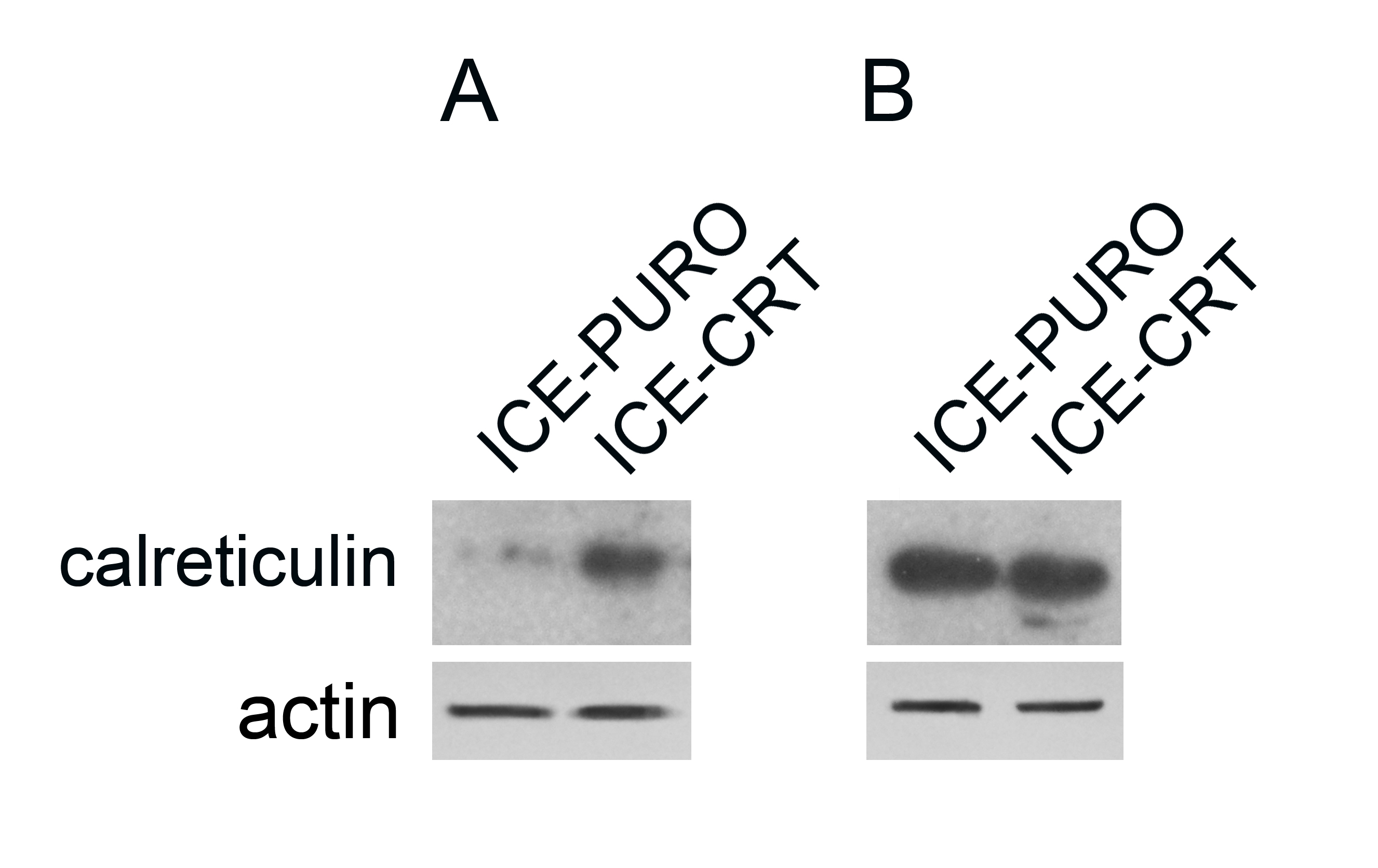

Supplement: Figure S3 — Detection of calreticulin by Western blotting. Cell lysates, equivalent to 20 μg of protein, were separated by SDS–PAGE and transferred onto nitrocellulose. (A) In ICE-PURO and ICE-CRT whole cell lysate with anti-calreticulin rabbit monoclonal antibodies-(D3E6) XP® Rabbit mAb #12238 (Cell signaling). (B) In ICE-PURO and ICE-CRT whole cell lysate with anti-calreticulin rabbit polyclonal antibody (ABIN361835) (Antibodies Online) which do not recognize porcine calreticulin. [file Image3.JPEG]

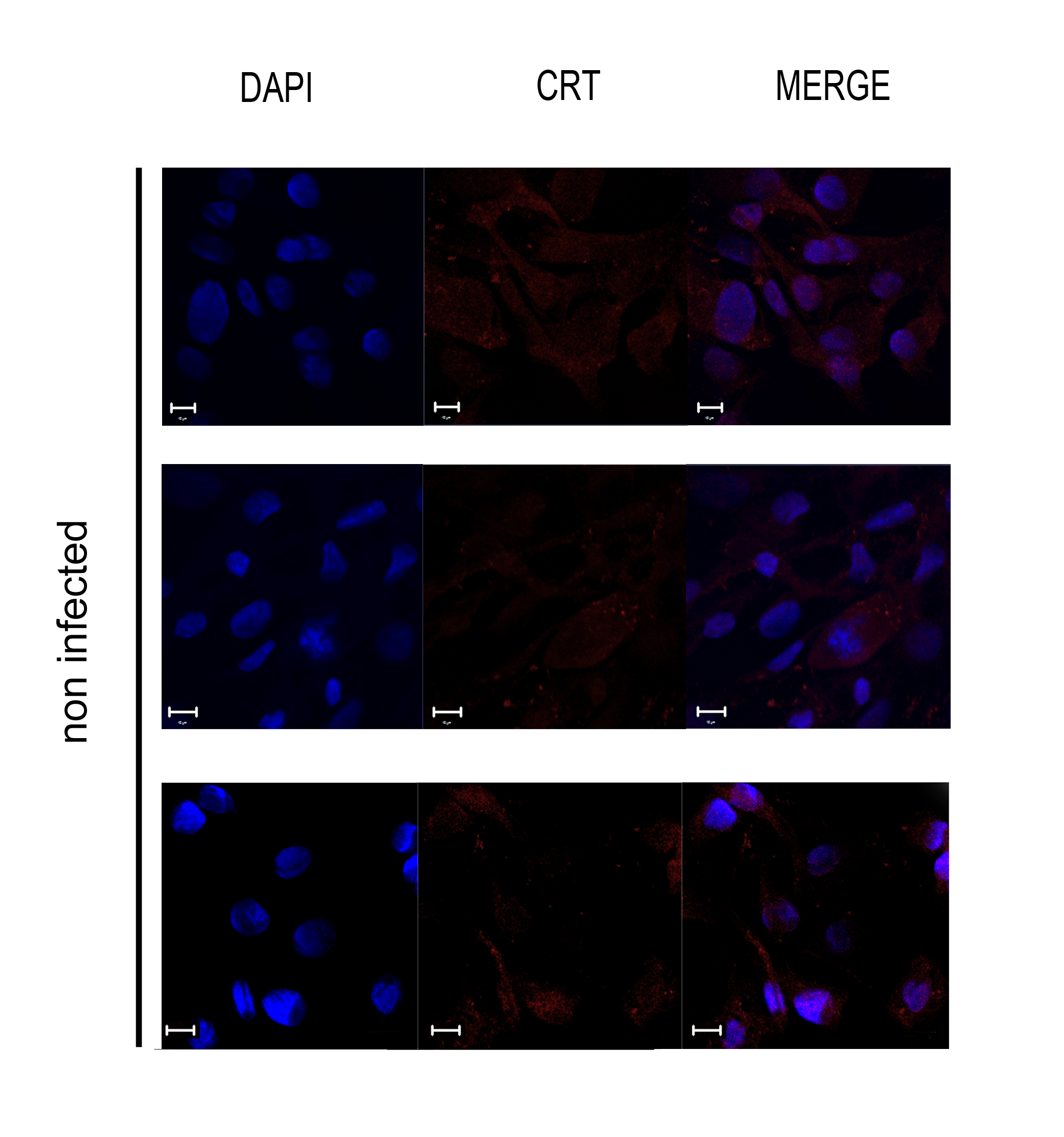

Supplement: Figure S4 — Localization and distribution of CRT in IPEC-J2 cells. Images were acquired on an LSM 510 META microscope (Carl Zeiss, GmbH Germany) using a PLAN-APOCHROMAT 63x/1.4 OIL DIC M27 objective. Image acquisition was performed using ZEN 2009 Light Edition software. Bars represent 10 μm. Membrane CRT organized in dot aggregates are indicated by arrows. [file Image4.JPEG]

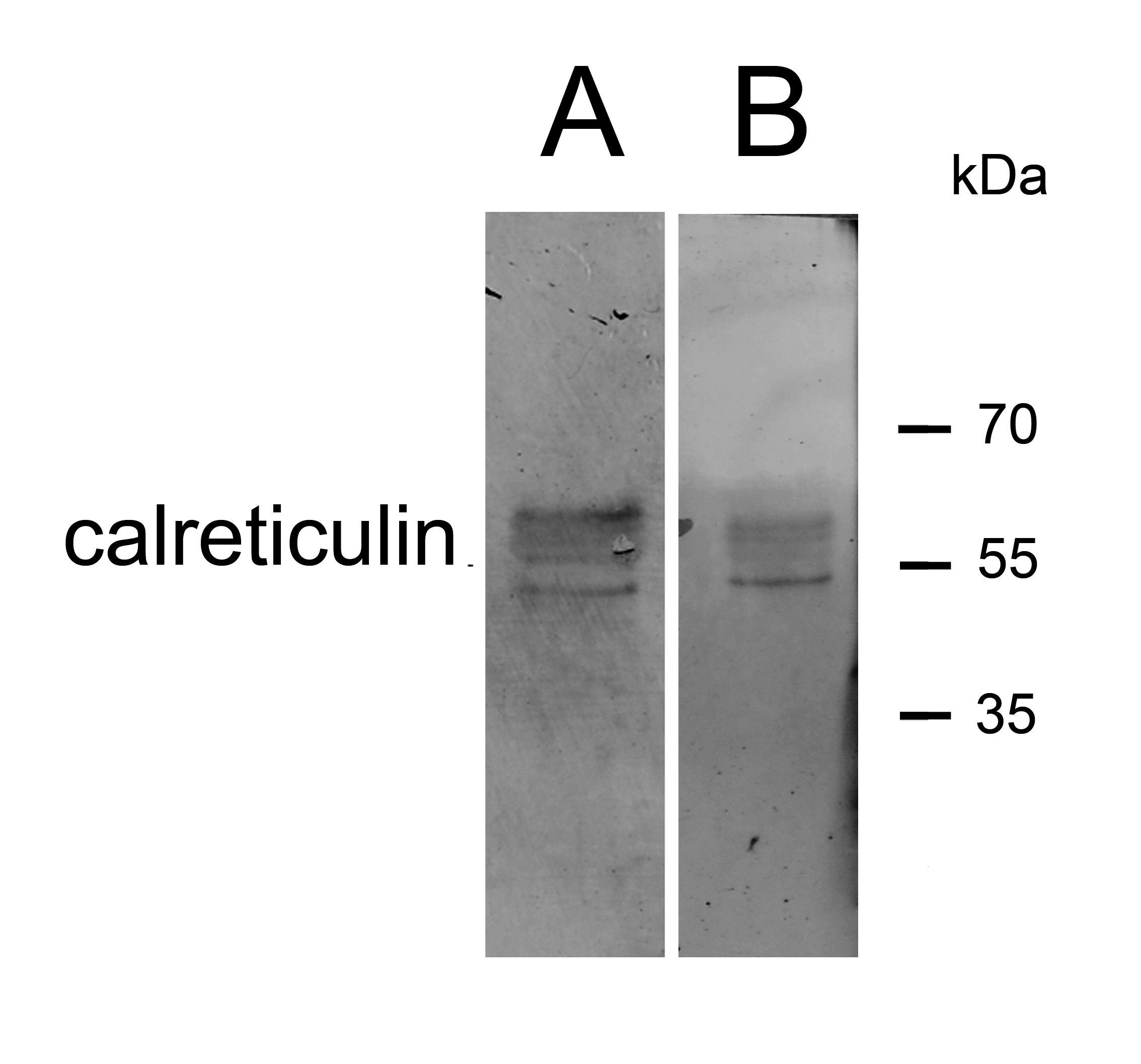

Supplement: Figure S5 — Interaction with recombinant porcine calreticulin. (A) Far-Western blotting analysis of FimH adhesin binding to recombinant porcine CRT. CRT (0.5 μg) was subjected to SDS–PAGE and transferred onto nitrocellulose. CFimH, C63FimH and EFimH were incubated with CRT immobilized on the membrane and then detected with anti-FimH rabbit polyclonal antibody and secondary anti-rabbit antibody. (B) Detection of recombinant calreticulin (0.5 μg) by Western blotting with anti-calreticulin rabbit monoclonal antibodies secondary anti-rabbit antibody. Protein was separated by SDS–PAGE and transferred onto nitrocellulose. [file Image5.JPEG]
